# Norepinephrine reuptake blockade to treat neurogenic orthostatic hypotension

**DOI:** 10.1007/s10286-021-00808-3

**Published:** 2021-05-11

**Authors:** Graeme Eisenhofer, David S. Goldstein

**Affiliations:** 1 Department of Medicine III, Institute of Clinical Chemistry and Laboratory Medicine, University Hospital Carl Gustav Carus, Technische Universität Dresden, Fetscherstrasse 74, 01307 Dresden, Germany; 2 Autonomic Medicine Section, Clinical Neurosciences Program, Division of Intramural Research, National Institute of Neurological Disorders and Stroke, National Institutes of Health, Bethesda, MD USA

**Keywords:** Supine hypertension, Atomoxetine, Ampreloxetine, Lightheadedness, Multiple system atrophy, Parkinson disease

Neurogenic orthostatic hypotension (nOH) is a not infrequent consequence of neurodegenerative disorders and can result in impaired mobility, falls and associated fractures, decreased quality of life, hospitalizations, and increased mortality. The disorder often coexists with supine hypertension, which carries additional cardiovascular risks and complicates the treatment of nOH. While non-pharmacological measures such as elevation of the head of the bed at night, taking frequent, small, snack-like meals, hydration, compression stockings, and lifestyle modifications are important in the clinical management of nOH, there has been growing emphasis on pharmacological treatments. These include the orally active α-adrenergic receptor agonist midodrine, the norepinephrine pro-drug droxidopa, the sodium-retaining mineralocorticoid fludrocortisone, the acetylcholinesterase inhibitor pyridostigmine, and sympathomimetic amines such as amphetamines [9].

In this issue of *Clinical Autonomic Research*, Lo and colleagues describe the pharmacokinetic and pharmacodynamic properties of ampreloxetine (TD-9855), a novel inhibitor of the cell membrane norepinephrine transporter (NET), as a potential new treatment for nOH, currently under clinical development by the pharmaceutical company Theravance Biopharma [6]. If clinical trials underway confirm the efficacy and safety of the drug, the agent would expand the pharmacotherapeutic armamentarium against nOH.

NET inhibitors for nOH is not a new concept, with other “oxetine” drugs, such as atomoxetine, being used [12, 13]. Of concern with these agents is the possibility of worsening supine hypertension. As Lo and colleagues report, ampreloxetine has a long plasma half-life of 30–40 h. This means that with daily dosing the levels of the drug would not plateau until after several days. Therefore, adverse effects might not appear immediately after initiating treatment. Blood pressure monitoring with attention to blood pressure at night will be key for patient safety.

In addition to the issue of supine hypertension, the underlying pathophysiology can guide the choice of pharmacotherapy for nOH. Lo and colleagues focused on synucleinopathies, which are disorders characterized by the abnormal deposition of the misfolded protein, α-synuclein, in central or peripheral neurons or glial cells. Synucleinopathies include Parkinson disease (PD), dementia with Lewy bodies (DLB), pure autonomic failure (PAF), and multiple system atrophy (MSA). These disorders typically feature baroreflex failure [5]. Consequently, treatment with drugs that enhance the delivery of endogenous norepinephrine to the adrenergic vascular receptors, such as NET inhibitors, should result in a sustained pressor response. This potentially amplifies concern about worsening supine hypertension with ampreloxetine.

Although there is some overlap in clinical presentations, the sites of the lesions in synucleinopathies vary, and in the study of Lo and colleagues the different disorders were lumped. Severe peripheral noradrenergic deficiency is a feature of PAF and PD with nOH but is rarely associated with MSA [3, 10]. Ampreloxetine therefore might be expected to produce larger, more sustained blood pressure increases in patients with preserved peripheral noradrenergic innervation (i.e., MSA) than in patients with marked peripheral noradrenergic deficiency (i.e., PD with nOH or PAF). This has already been shown for atomoxetine, which substantially increases blood pressure in patients with MSA but not that much in PAF [13]. Interestingly, the converse is observed with droxidopa, which produces a more pronounced blood pressure increase in patients with marked peripheral noradrenergic deficiency, as measured by low plasma norepinephrine levels [11]. Studies involving ampreloxetine and other nOH drugs should therefore consider the nature of the pre- and post-ganglionic lesions. Indeed, in their report, Lo and colleagues indicate that such considerations are being explored in the ampreloxetine clinical trials underway.

Tricyclic antidepressants, which are classical blockers of the NET, cause orthostatic hypotension [8] and are contraindicated in patients with nOH [9]. This adverse reaction results from sympathoinhibition [1], a centrally mediated effect that opposes the impact of peripheral NET blockade to increase norepinephrine overflow from sites of release. It would be informative to learn whether ampreloxetine alters the rate of post-ganglionic sympathetic nerve traffic, since if the neuronal outflow were decreased this might offset effects of reuptake inhibition at sympathetic nerves on delivery of norepinephrine to its receptors. To document that ampreloxetine actually does inhibit NET function, Lo and colleagues used a neurochemical approach based on concurrent measurements of plasma norepinephrine and 3,4-dihydroxyphenylglycol (DHPG), the main intra-neuronal metabolite of norepinephrine. A common flaw of such strategies, as is evident in the study of Lo and colleagues, is that under resting conditions most circulating DHPG is not derived from neuronal reuptake of norepinephrine but from enzymatic deamination of norepinephrine leaking passively from storage vesicles into the cytoplasm [2]. DHPG production reflects the size of sympathoneural stores of norepinephrine more than the release and reuptake of the transmitter. This means that plasma DHPG concentrations depend on the intactness of sympathetic innervation. Moreover, complicating the matter even further, in PAF plasma DHPG concentrations are increased with respect to norepinephrine [4], possibly because of a vesicular storage defect in sympathetic nerves. To employ measurements of DHPG for the assessment of the actions of certain drugs on the NET it is critical to measure changes in plasma DHPG relative to those of norepinephrine during changes in sympathetic outflow, such as evoked by orthostasis [2]. There are, however, pitfalls to this approach in patients with nOH due to impaired or absent sympathoneural responses to orthostasis. In such patients, increases in plasma norepinephrine relative to DHPG during orthostasis can reflect decreased cardiac output and consequently prolonged circulatory clearance of analytes [7] and, therefore may not be useful for assessing NET function.

An alternative approach for examining NET function, which would be more straightforward than the neurochemical approach, involves use of radiolabeled ligands such as ^11^C-methylreboxetine for NET positron emission tomography (PET) imaging in the heart and brain. The NET inhibitor, methylphenidate, competes with ^11^C-methylreboxetine for NET sites in the human brain [10]. This approach, which was already used to assess the CNS actions of ampreloxetine [14], could be re-used to assess its peripheral action on NET function.

In summary, while the results of this clinical trial are promising, the risk/benefit analysis of ampreloxetine to treat nOH remains to be fully defined. Large placebo-controlled clinical trials are underway to do so. There are substantial complexities inherent with drugs that inhibit function of NETs concurrently in the brain and periphery. Although a decreased plasma DHPG/NE ratio is consistent with NET inhibition, this ratio is complexly determined, especially in disorders that involve central and peripheral noradrenergic deficiency. Since responses to ampreloxetine could depend crucially on whether there is peripheral noradrenergic deficiency, careful consideration should be given to the possibility of differences in therapeutic effectiveness according to the nature of the lesions affecting the sympathetic nervous system.
